# Shape Memory Alloy-Based Wearables: A Review, and Conceptual Frameworks on HCI and HRI in Industry 4.0

**DOI:** 10.3390/s22186802

**Published:** 2022-09-08

**Authors:** Rupal Srivastava, Saeed Hamood Alsamhi, Niall Murray, Declan Devine

**Affiliations:** 1 Confirm Center for Smart Manufacturing, Science Foundation Ireland, V94 C928 Limerick, Ireland; 2 PRISM Research Institute, Technological University of the Shannon, Midlands Midwest, Athlone, N37 HD68 Co. Westmeath, Ireland; 3 Department of Electrical Engineering, Faculty of Engineering, IBB University, Ibb 70270, Yemen; 4 Department of Computer and Software Engineering, Technological University of the Shannon, Midlands Midwest, Athlone, N37 HD68 Co. Westmeath, Ireland

**Keywords:** shape memory alloys, smart wearables, hand exoskeletons, human-robot interaction, human-computer interaction

## Abstract

Ever since its discovery, the applications of Shape Memory Alloys (SMA) can be found across a range of application domains, from structural design to medical technology. This is based upon the unique and inherent characteristics such as thermal Shape Memory Effect (SME) and Superelasticity (or Pseudoelasticity). While thermal SME is used for shape morphing applications wherein temperature change can govern the shape and dimension of the SMA, Superelasticity allows the alloy to withstand a comparatively very high magnitude of loads without undergoing plastic deformation at higher temperatures. These unique properties in wearables have revolutionized the field, and from fabrics to exoskeletons, SMA has found its place in robotics and cobotics. This review article focuses on the most recent research work in the field of SMA-based smart wearables paired with robotic applications for human-robot interaction. The literature is categorized based on SMA property incorporated and on actuator or sensor-based concept. Further, use-cases or conceptual frameworks for SMA fiber in fabric for ‘*Smart Jacket*’ and SMA springs in the shoe soles for ‘*Smart Shoes*’ are proposed. The conceptual frameworks are built upon existing technologies; however, their utility in a smart factory concept is emphasized, and algorithms to achieve the same are proposed. The integration of the two concepts with the Industrial Internet of Things (IIoT) is discussed, specifically regarding minimizing hazards for the worker/user in Industry 5.0. The article aims to propel a discussion regarding the multi-faceted applications of SMAs in human-robot interaction and Industry 5.0. Furthermore, the challenges and the limitations of the smart alloy and the technological barriers restricting the growth of SMA applications in the field of smart wearables are observed and elaborated.

## 1. Introduction

The exponential advancement in cobotics, human-integrated robotics, and human-computer interaction require affordable, lightweight, and efficient smart materials. High-performing intelligent systems which can integrate humans in real-time Human-Computer Interaction (HCI), thus expanding the reach of human capacity one step further, are the need of the hour. Even though the concept of human-machine integration might seem a more recent development, the research in the field can be dated back to the 1970s. Reddy [1], compiled the recent research in the field of speech recognition by machines and how human-machine interaction will revolutionize future technologies. In the early 1970s, a group of graduate physics students at the University of California SC started working on the concept of a concealed computer in a shoe to play roulette and completed the project in 1978 with a considerable profit. This work, and the group which called themselves Eudaemons, was later mentioned by Bass [2] in his book ‘The Eudaemonic Pie’. The idea of integrating machines with our everyday objects has been around for a long while; however, a practical application came along in 1980, with Bolt’s [3] work on the integration of graphics interface with voice and gesture-based commands. Since then, a tireless effort to integrate humans directly with the machines has been ongoing, and each passing year comes with a smarter watch [4], smarter home [5], helping monitor human health and day-to-day requirements. Not only are these wearables helping the user coordinate with the environment, but the smart ecosystem thus formed is helping keep track of the user’s health and requirements. This creates two-way assistance between the user and the environment, thus evolving the Internet of Things (IoT) into the Internet of Wearable Things (IoWT). The ease of access and portability of these smart wearables play a significant role in defining the useability and popularity of the product [6].

In recent years, the usage of smart materials in various technical fields, especially healthcare and IoT, has exploded [7]. This is being achieved by introducing smart materials such as wearable sensors in wearable technology, which are then connected to the ecosystem of multiple user-interface devices, alarming systems, and other general electronic devices connected to the cloud. Not only is this concept being used for biofeedback data to analyze and monitor the user’s health conditions, but it is also being used to create a data set of environmental conditions of the user and identify hazardous zones [8]. Smart materials, such as piezoelectric materials, have a notable role in developing healthcare wearable devices, including piezopolymers, piezoceramics, piezoelectric semiconductor materials [9], and soft piezoelectric materials for sensing [10]. However, their applications in the field of smart wearables are limited and contained to research labs and not commercialized due to the expense and usability of the piezoelectric materials. Another fascinating upcoming field in the domain of smart wearables is flexible [11] and stretchable electronics [12] for their use in wearable devices and garments [13,14]. Most of the research that has found its commercial application mainly falls under healthcare [15] and patient monitoring [16,17]. This helps early detection of diseases, especially during the asymptomatic stage. From biochips identifying illnesses [18] to self-adhesives for the skin [19], the healthcare wearable research is expanding its boundaries from simple heart monitors and pedometers. In similar research, the Cyrcadia breast monitor is helping detect breast health for early signs of breast cancer through adhesive patches recording thermodynamic metabolic data [20].

Shape Memory Alloys (SMAs) are smart materials with unique characteristics caused due to temperature- and stress-induced phase transformation. Although SMAs provide exciting opportunities to create a new sensor and actuator mechanism, their practical use is complicated by their extremely nonlinear behavior and heat activation requirements. However, these SMA-based actuation mechanisms, which focus on soft robotics, offer qualities that make them an enticing alternative. SMAs’ versatility allows us to create actuation components in various topologies and forms (e.g., helical springs, torsion springs, straight wires, cantilever strips, and torsion tubes), allowing them to be used in a variety of applications. Despite these benefits, SMAs have drawbacks that must be considered when designing actuator systems, such as low actuation frequency, complicated controllability, and low energy efficiency. These challenges arise when working with the shape memory effect caused due to thermal actuation. At the same time, the applications utilizing the pseudoelastic behavior are far less complex, highly controllable, and do not require any energy consumption. Previous literature reviews in the field of SMAs and their applications in the field of wearables have been limited to their use in soft robotics [21] and medical devices [22].

In this paper, the use cases that work on the shape memory effect and pseudoelastic property are presented with a focus on their sensing applications. By capturing the shape morphing caused due to the shape memory effect using strain sensors, as discussed in the coming sections, the SMAs contribute to the overall sensing mechanism in the discussed use cases. A similar discussion on using the shape memory effect for an overall sensing mechanism has been done by Tung et al. [23]. The following section discusses the motivation for the proposed concepts and the significant contribution of the use cases for advancing research in human-computer and human-robot interactions.

## 2. Motivation and Contributions

The use of the shape memory effect and pseudoelastic characteristics of the SMAs in combination or individually allow myriad applications. SMAs are helping overcome many challenges in smart wearables for assistance, rehabilitation, and protection. However, the use of these materials is limited and commercial applications in wearables are yet to be achieved. This is mainly due to the low actuation frequency of the SMAs and the requirement of thermal actuation to attain the shape memory effect. The use case or the conceptual framework presented in this paper utilizes the pseudoelastic property of SMA.

The primary requirement of any wearable smart material is its elasticity, flexibility, and fatigue life, dependent on the former. Furthermore, one needs to reduce the electrical input required to the minimum as the smart wearable is susceptible to water splashes and will be in touch with the user’s skin for long periods. Once embedded within wearables, smart materials cannot frequently undergo maintenance, which is a cumbersome task. SMA fibers, wires, or springs, when in their Austenite phase, can withstand high load and high elastic deformation without deforming plastically. They also have a higher fatigue life than other metallic alloys available. These qualities make SMAs an ideal candidate to be used as an embedded material in wearables more inclined to extreme deformations and rough use. The conventional pressure sensors require a high impedance circuit and are either susceptible to vibration or cannot measure solid-state pressure, such as piezoelectric sensors. Hence, considering the unique characteristics of SMA as discussed and the several challenges faced by the conventional temperature and pressure sensors, the use of SMA fibers and SMA springs is proposed in *smart jackets* and *smart shoes*, respectively. While in the former case, the response to change in temperature is extracted, the spring deformation is measured in the latter case. The obtained data is then utilized for smart environment control and follower leader robot and integrated with the IoT for smart homes and smart industries applications.

The main contributions through our proposed framework are as follows:

1. The authors compile the history and state-of-the-art literature focused on the field of SMA-based smart wearables, and the findings are converged on the challenges faced while using SMAs in wearable applications. To the authors’ knowledge, a survey focusing on SMA-based wearables has not been conducted previously.
2. The primary questions- how can SMA be used in wearables, and how to overcome the material behavior-related challenges are discussed. Thereby, two conceptual frameworks of SMA-based smart wearables for Human-Computer and Human-Robot Interaction in a *smart factory* environment are introduced.
3. In the first use case, SMA in smart clothing, i.e., *smart jacket* for HCI, is presented. In this use case, the focus is on capturing the thermal variation response of the embedded SMA fibers and filtering this data to obtain commands for the heating/cooling of the room/warehouse in harsh or hazardous conditions. This data is obtained from multiple users, and a collaborative approach to optimize the instructions is proposed.
4. In the second use case, SMA-based wearables for Human-Robots Interaction (HRI) in Industry 5.0 are presented. Smart Shoes with soles embedded with SMA springs are conceptualized for Follower-Leader robot technology and humanoid motion training. These springs are also suggested to be ergonomically beneficial for the user by providing higher suspension in the shoes. The movement behavior of the user is analyzed based on the amount and type of load. This data is then fed in sitting-walking formats for the robot to learn and mimic the pattern. Both the use cases are finally discussed as a part of the Industrial Internet of Wearable Things (IIoWT) for creating a safer working environment.

**Figure 1:**
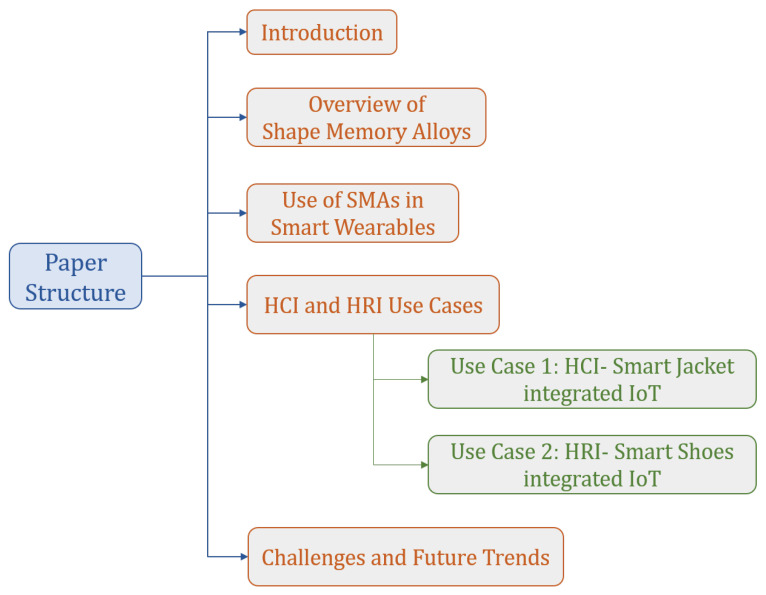
Paper Structure. The importance of adding smart wearables to IoT is discussed in Section 5.

The article structure is outlined in Figure 1. The article begins with an overview of SMA and their myriad of applications in Section 3, followed by a focus on their applicability in the field of smart wearables in Section 4. Then the details move on to the two proposed conceptual frameworks in Section 5, where the integration of the smart products with IoT and their benefits in Industry 4.0 (I4.0) is emphasized. Finally, in the last section, Section 6.2, the challenges the proposed use cases have to offer are discussed. The authors also discuss how to overcome these challenges and promote the applicability of these smart products in social distancing, isolation, and remote working during similar to the COVID-19 pandemic is examined and presented.

The articles selected for the review part of the paper are based on the earliest work and recent work in the field. The Impact Factor (I.F.) of the journals has also been taken into consideration, and most articles are from the journal with I.F. greater than 2, unless the citations of the article are high enough not to consider the impact factor. The keywords selected for the literature review were *Shape Memory Alloys*, *Smart Wearables*, *Internet of Things*, *SMA-based wearables*, *and Follower-Leader Robots*.

## 3. Overview of Shape Memory Alloys

The term ‘shape memory’ is coined due to the unique ability of some metallic alloys and some polymers to remember a parent shape. These metallic alloys, upon thermal actuation, transform from their low-temperature martensite phase to the high-temperature austenite phase. During this transformation, the alloys recover the imparted deformation through loading and return to their pre-defined and pre-trained parent shape. This process is called the Thermal Shape Memory Effect (SME). SME in-shape memory alloys can be observed either as a One-Way Shape Memory Effect (OWSME) or a Two-Way Shape Memory Effect (TWSME). These two effects can be created and optimized by controlling the heat treatment of the SMA. In the case of OWSME, the SME can be observed by deforming the SMA in its martensite phase and then heating it to undergo a phase transformation from martensite to parent austenite phase, thus recovering its shape during the process. Whereas in the case of TWSME, the SMA can be trained to remember two shapes, one at lower temperature martensite phase and another at higher temperature austenite phase. Thus, the SMA can be deformed, and two different shapes can be extracted based on the heating or cooling of the alloy.

**Figure 2:**
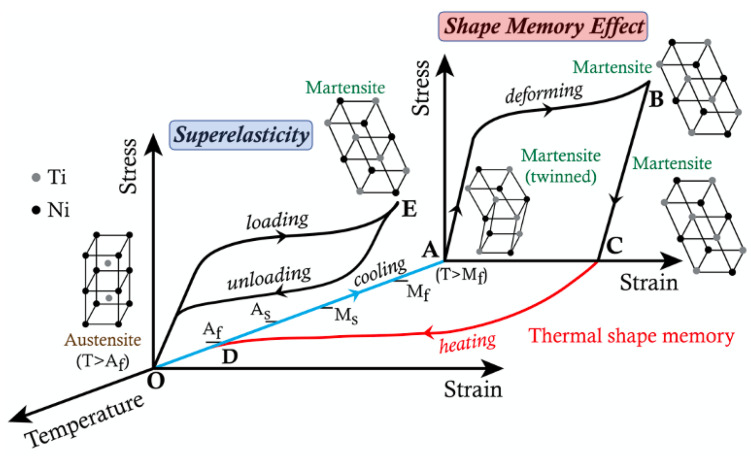
Stress-Strain-Temperature diagram of shape memory alloys showing Shape Memory Effect and Superelasticity or Pseudoelasticity.

Another attractive property these alloys exhibit is the ‘superelasticity’ or ‘pseudoelasticity’. This property allows the material to undergo a huge amount of stress and deformation without permanent deformation and snap-back to its original shape and size. Both these properties are explored and utilized based on the application requirement. While SME is usually taken advantage of when shape morphing is desired, and thermal environment is not a constraint [24], the ‘superelasticity’ is beneficial where the structure is subjected to high deformation, wear, and tear [25]. SME was first discovered in the Au-Cd (Gold-Cadmium) alloy by Chang and Read [26]; however, the effect gained popularity after the discovery of SME in Nickel-Titanium alloys by Buehler et al. [27]. Termed NiTiNOL (Naval Ordinance Lab), this alloy demonstrated a more significant shape memory effect than other shape-memory materials. Not only a 100% recovery of up to 8% strain was observed, but a more than two-fold increase in Young’s modulus in the austenite phase in comparison with the martensite phase was also seen. Figure 2 shows SMA’s qualitative stress-strain behavior with respect to temperature.

Continuous growth in active control of structures and their properties is giving rise to a new class of synthetic adaptive composites. These characteristics, such as variable flexibility, help in customizing the static and dynamic responses of a structure. One way to achieve these variable responses is by adjusting the ply layup order, thickness of plies, fiber orientation, and volume fraction. Combining the benefits of heterogeneous current conventional composites with self-adapting and actively controllable reinforcing constituents, we get a new domain of active composites. These embedded active constituents can be smart materials, such as SMA, piezoelectric fibers, and magnetostrictive nano-particulates. As a result, we get functional composites with special properties such as self-healing, vibration attenuation, damage sensing, and shape morphing. Bachman et al. [28] by embedding SMA wires and shunted piezoelectric material, investigated the enhancement of passive damping of Carbon Fiber-Reinforced composites (CFRP); Arrieta [29] designed piezoelectric composites to explore a bi-stable wing-shaped model.

SMAs for the structural control of composites can be achieved by SMA fiber reinforcement or by integrating the material externally, for example, through bonded sleeves. An offset SMA fiber reinforcement in the composite can result in shape morphing and structural damping upon thermal actuation of the SMA. The concept behind it, as discussed previously in this section, is the phase transformation of the SMA from martensite to austenite and vice-versa when thermally-induced or stress-induced actuation. Loughlan et al. [30] explored laminated composites with SMA actuators within sleeves along the neutral plane for active buckling control; Machairas et al. [24] investigated an adaptive airfoil with fluid-structure interaction actuated by a two-link adaptive mechanism integrated into SMA wires. Recently, automotive fender skirts that can morph to reduce drag utilize SMA integrated through ribs [31].

SMA integrated carbon nanotubes (CNT), another unique category of smart composites, were investigated by Fonseca et al. [32]. The authors manufactured shape memory polyurethanes (SMPU)-integrated carbon nanotube (CNT) nanocomposites through mechanical melt mixing and polymerization. An exploration in the field of SMA in medical applications has led to successful SMA-based products, such as SMA bone scaffolds by Bormann et al. [33] for bio-compatible load-bearing, SMA stents [25], and most popularly used- SMA dentures [34]. The advancement of soft robotics further led to the increased popularity of SMA springs which have a more significant displacement in comparison to SMA wires. Holschuh and Newman [35] found its implementation in morphing aerospace systems with low spring index SMA coils. Narayanan and Elahinia [36] proposed a combined bias spring-SMA wire actuator rotary manipulator, and Thomas et al. [37] investigated optimal dimensioning and maximizing the stroke of the SMA bias-spring linear actuator model.

**Figure 3:**
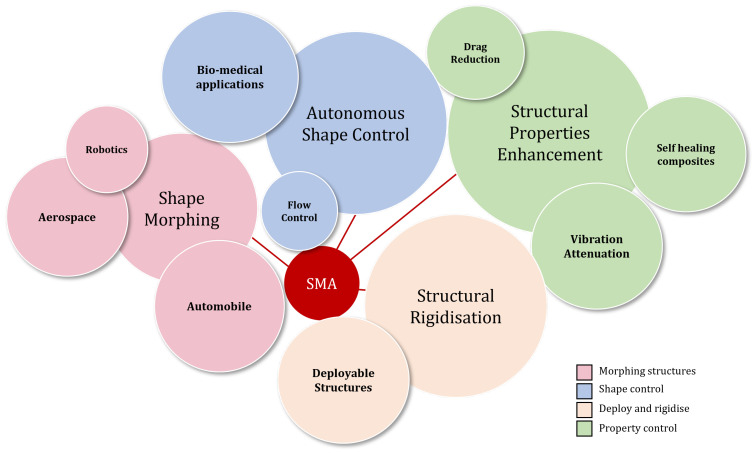
Major application domain of Shape Memory Alloys. The Superelastic property finds more commercial applications as compared to the Thermal Shape Memory Effect. The use of SMA in smart wearables is currently in its laboratory and experimental phase.

Bartlett et al. [38] conducted a study on SMA strips functioning as bistable arches for wing chord morphing. Another application for SMA strips is in nanotechnology as tunable resonators [39]. Similarly, tunable metamaterial beams with shifting elastic moduli due to phase transformation are designed to enable active bandgap shifting over a temperature range [40]. SMA embedded fiber-reinforced composites have been deeply explored by the academic community [41]. As discussed before, it is proven by prior research that SMA embedded FRPs can show active and passive damping, enhancement of impact [42] and buckling behavior, cause shape morphing [43,44], and provide self-healing properties [45]. In the textile industry, SMA fibers are giving rise to morphing and self-fitting wearables [46,47]. Lelieveld et al. [48] devised a combined Shape Memory Polymer (SMP) and SMA wire smart composite achieving 90${}^{\circ }$ bending deformation. A general outline of the application domain of the SMAs is summarised in Figure 3. A substantial amount of research in this field can be summarised into two major categories: Active Property Tuning (APT) and Active Strain Energy Tuning (ASET) to modify the stiffness of the hybrid composite and for shape recovery purposes, respectively [49]. APT is a method of modifying the effective stiffness of the composite using SMA residual and high actuation stresses. Some work has been done by Yang Hua et al. [50], experimenting with the Effective negative Coefficient of Thermal Expansion (ECTE) of fibers like carbon fiber or Kevlar to attain structural stability over a given temperature range. A similar strategy which was earlier explored using the negative ECTE of SMA by Baz and Chen [51] was utilized to improve the overall performance of drive shafts embedded with SMA. The shape morphing behavior without compromising the structural stiffness is realized through the continuous embedding of SMA in flexible beams [52], rods [53], and cylinder and pressure vessels [54].

Similarly, active and passive vibration control is also obtained through the strategic embedding of SMA in various composite structures [55,56,57]. For example, Lau [58] investigates the frequency response of SMA embedded composites over a temperature range and introduces a theoretical model to capture this behavior. Damping is another reason for vibration control-based studies; Baburaj and Matsuzaki [59] discuss their findings on numerical studies of specific damping capacity of SMA laminated composite plates. Apart from property tuning and shape morphing applications, SMA-embedded composites are also used for damage suppression and health monitoring purposes. Kuang and Cantwell [60] obtained localized SMA actuation detecting impact damage in CFRP, and Saeedi and Shokrieh [61] experimentally studied the effect of pre-strained SMA on fracture behavior of the polymer at different temperatures and with varying SMA diameter.

Kirkby et al. [62], in their work, demonstrated this behavior by combining a microencapsulated liquid healing agent and SMA wires wherein the SMA is actuated to pull the crack together and bind it using the healing agent. In a more recent work by Karimi et al. [63], the thermomechanical response of SMA is converted to traction-separation response, thus presenting a numerical model for the self-healing behavior of SMA embedded composites. Finding the utility of SMA as a sensor has opened new doors of exploration in structural health monitoring. Nagai and Oishi [64] find a correlation between the strain in their composite and the variation of electrical resistance in the embedded SMA wire, thus estimating the material damage indirectly. Soon after this experimental work, Cui et al. [65] proposed a mathematical model capturing the sensing function of the embedded SMA for structural health monitoring. A nonlinear finite element model by Khalili et al. [66] solves the dynamic analysis of an SMA-embedded composite plate. Hence, a well-formed mathematical foundation supports the various fields of applications of SMAs.

## 4. SMA in Smart Wearables

**Figure 4:**
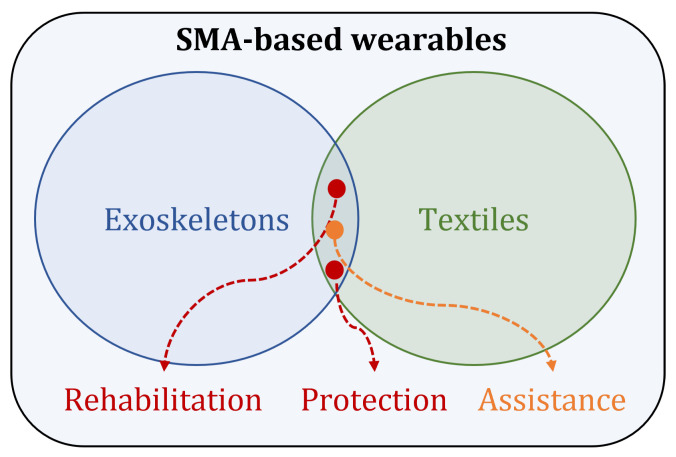
The major application domain of SMA-based wearables can be found in exoskeletons and textiles for either rehabilitation or protection.

Given the versatility of memory alloys, they have been extensively explored for applications in wearable technology. We can broadly categorize these applications into two domains-exoskeletons and textiles and their purpose as rehabilitation or protection, as seen in Figure 4. Using shape memory alloys as actuators have been around for a long time. In their work, Hirose et al. [67] discussed micro-actuators driven by the shape memory effect and proposed a design configuration called the ζ-array for the SMA actuator. This prototype will further become the foundation of integrating shape memory alloys’ thermal shape memory effect with robotics and other mechanisms. These days, the applications of SMAs in mechanical engineering, robotics, and aerospace engineering are revolutionizing the field by making the system smarter. From being embedded inside fiber-reinforced composites for shape morphing [68,69] and vibration control applications [70], to be used in dentistry [71] and as the building material of the structures [72], SMAs have now become an integral part of smart structures and systems. Taking the concept further, SMAs are now integrated with textiles and exoskeletons for protection, safety, and rehabilitation purposes.

The bio-compatibility of NiTiNOL, one of the most popular SMA, led to an exponential increase in the research area of SMA-based wearables. The earliest work was done by Laurentis et al. [73], where they fabricated an SMA-actuated prosthetic hand using a rapid prototyping technique. Due to the gap in technology at that time, Price et al. [74] discussed the issues related to design, control, and instrumentation in practically realizing the application of SMAs as artificial muscle for a robot hand. Another work in this field was a new biomimetic wearable prosthetic robotic hand based on tendon-driven actuation [75]. A soft orthotic prototype was designed and fabricated by Stirling et al. [76] for corrective gait treatments using actively controlled SMA springs; however, the low frequency of actuation of the SMA springs acted as a major challenge.

More recently, Hwang et al. [77] developed an SMA-based miniature haptic ring for cutaneous force feedback. The ring can display touch/pressure and shearing force on the user’s fingerpad. Similar work on an SMA-based haptic ring was done by Chernyshov et al. [78], where instead of using the SMA wires as actuators for vertically movable parts, they use SMA wire rings encapsulated inside silicon tubes, thus causing the restricting motion or the force feedback to the user. Some notable recent works done incorporating SMA in exoskeletons for rehabilitation purposes include a hand neuro-rehabilitation system [79], tendon-driven hand exoskeleton [80], soft wearable robot for assisting wrist motion [81], and elbow exoskeleton for rehabilitation therapy and patient evaluation [82].

**Figure 5:**
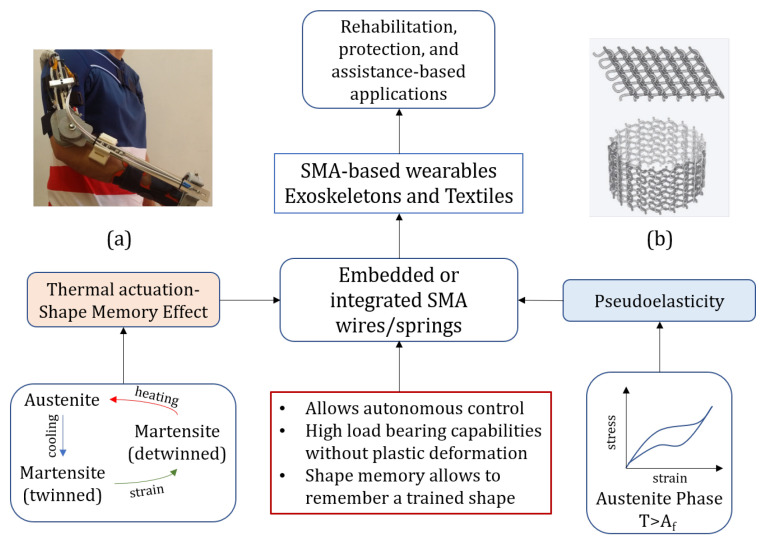
The SME and the Pseudoelasticity or Superelasticity are the properties that make SMA-based wearables an improved version of existing smart wearables. (**a**) Tendon driven elbow exoskeleton for joints rehabilitation [85], (**b**) SMA knit fabric for self fitting garment [47].

There are many applications of SMAs in wearables, including SMA-based textiles wherein the SMA wires are either woven or embedded as fibers in the clothing. Most of these applications come with the idea of providing safety and customizability to the user; however, Holschuh and Newman [83] developed compression garments for astronauts to assist them in extravehicular activities and during re-entry and landing. Park and Park’s work in the assistive technology application of SMA-based wearables presents an SMA-based suit-type wearable robot to enhance the muscular strength of the user, thus assisting it in day-to-day tasks [84]. Granberry et al. [47], present a temperature-responsive, self-fitting garment that envisions unpowered and automated fits in futuristic wearable technology. One of the use cases presented in this paper pertains to similar wearable technology in garments later in the paper. Table 1 compares the current work in the field of SMA-based wearables and the presented use cases, and Figure 5 shows the application of the two significant SMA properties in smart wearables.

## 5. HCI and HRI Use Cases

### 5.1. Use Case 1: Human-Computer Interaction: Smart Jacket

As we go deeper and broader into establishing an intelligent environment, from smart homes to smart healthcare and cities, we further sophisticate the fundamental units. The requirement for a smart environment is for the units to interact with the environment and each other, and to be equipped with features that enable them to integrate seamlessly and collaborate. Smartwatches, smart goggles, and smartphones are some of the many tools humans use in their day-to-day lives to interact with computers and synchronize their requisites with their surroundings. Mozer [86], one of the pioneers in the field, proposed the idea of a *Neural Network House* where the house ‘*essentially programs itself by observing the lifestyle and desires of the inhabitants and learning to anticipate and accommodate their needs*’. Ever since then, several conceptual frameworks for smart homes [87], factories [88], healthcare systems [89], and cities [90] have been devised which utilize deep-reinforcement learning and machine learning to create a smart environment.

Here, we propose the conceptual framework for a *smart jacket* interacting between the environment and multi-users in a defined domain. The idea for the *smart jacket* was conceived based on the ongoing research in field of SMA-based assistance textiles [91], spacesuits [83], and protective garments [92]. This research has shown successful applications in the required domain. Much research on transition temperature ’tuning’ of SMA has been conducted [93,94]; hence the working temperature range of the SMA can be based upon the environment and weather conditions. Since the phase transformation of other wearable fibers such as cotton and polyester is beyond human functional range, therefore, there will not be any interference with the phase transformation of SMA fibers.

**Figure 6:**
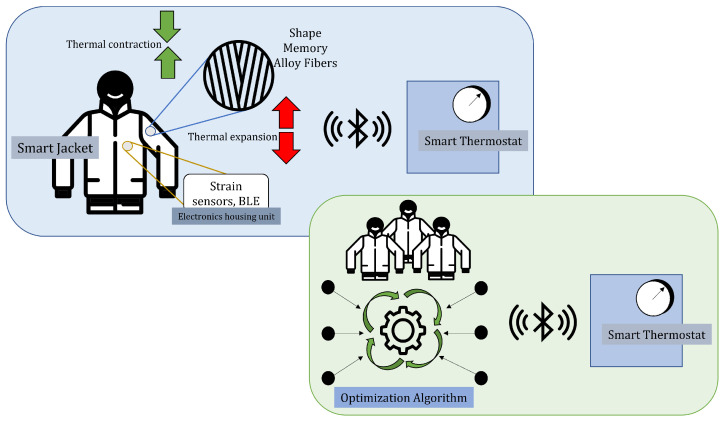
Human-Computer Interaction Use Case: Smart Jacket. Conditions showing single and multi-user states. The change in the length of the SMA fibers woven with the jacket is caused due to phase transformation caused due to a user temperature and environment temperature gradient.

**Figure 7:**
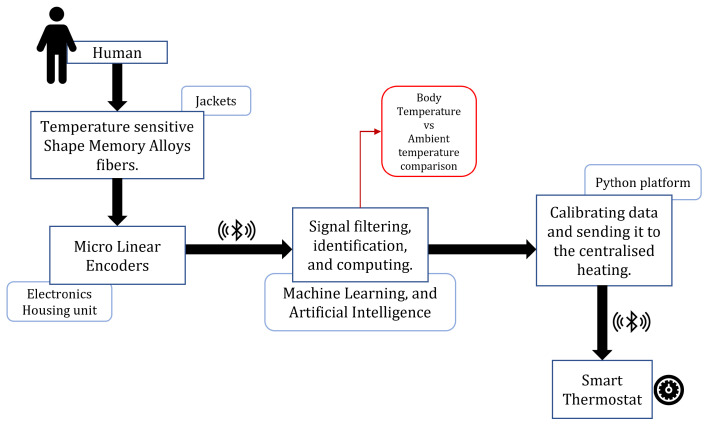
Algorithm for the HCI use case framework. The Machine Learning algorithm is essential to analyze and optimize the thermostat, especially in the multi-user case.

Figure 6 shows the concept for the shape memory alloy fiber-reinforced *smart jacket* that responds to the change in the body and the ambient temperature. As discussed in Section 3, the SMA can be programmed to morph its shape upon thermal actuation. Using this property, we reinforce the *smart jacket* with SMA fibers with actuation temperatures close to the ambient temperature or suitable for comfortable living conditions. Upon a fall or rise in temperature considerably above the defined thresholds, the change in the length of the SMA fibers is observed. The thermal actuation is caused due to the thermal gradient between the user and the environment, and no external thermal actuation, such as Joule heating, is provided to the SMA fibers. A change in thermal gradient leads to the phase transformation in the SMA fibers, and hence contraction and expansion will be observed as the SMA fibers are memory trained to behave in this manner. This expansion-contraction behavior is measured through embedded strain sensors or linear position displacement sensors, and the data is sent to the smart thermostat via Bluetooth. One end of the SMA fiber is fixed in the jacket, and the linear position sensors are directly attached to the other end of the SMA fiber to capture this contraction. It is to note that the placement of the SMA fibers in the jacket is designed to be on the front and back, thus removing any strains experienced due to limb movement or human motion. The smart thermostat is pre-programmed and trained to change its performance based on the data received from the users wearing the *smart jacket*. The signal or data received is filtered, identified, and computed before it is calibrated with the thermostat to decide the ambient room conditions. The proposed plan of action and the algorithm are shown in detail in Figure 7.

In a collaborative environment with multiple users, the data received from all jackets in use is compiled, and an optimized signal is sent to the thermostat. The input variables for the optimization will be the strain sensor data from each jacket, compared against standard stain sensor data responsible for near-perfect room temperature. A more prominent contrast between the strain sensor data between the user implies that one or more of the users have entered the room from outside. Thus, sudden sharp differences in thermostat temperature can be employed to maintain a temperature to reach the comfort level as soon as possible. This also allows the users to modify their requirements based on the environment, weather, and location. The data from the user in close vicinity to the closed domain is also received, which is then computed to smartly understand the weather conditions outside and optimize the thermostat if need be. The framework aims not only at creating a more organic method of maintaining comfortable conditions but also to find a sustainable solution to energy conservation. The body temperature recognition thus allows for the on/off conditions of the thermostat and maintains an economical and eco-friendly thermostat state.

In a hazardous environment prone to sudden increases or decreases in environmental temperatures, the *smart jacket* can act as an alarming device to send data to the required systems and maintain the room/chamber temperature. A smaller variation in the user and environment temperature can be used for a balanced working condition. In contrast, a more significant gradient between the user and environment temperature can either shut down the system or send data to boost the system to achieve a balanced environment condition as soon as possible. Combined with the IoT, this data can be used to analyze the working conditions and investigate hazardous zones and safe working zones, marking them accordingly. Furthermore, the data can be compiled and used to create a database to identify risk zones in the future and help enable a safer environment. The significant advantage of using SMA material instead of any other smart material is the ease of embedding SMA fibers as a textile for the *smart jacket*. Also, the presented use-case SMA fibers do not require any actuation apart from the difference between the user temperature and room temperature to change its shape and send this shape change data to strain sensors. This eliminates an AC power supply to the *smart jacket*, reducing any electrocution-based risk and making the jacket highly portable. Hence, SMA fibers, highly sensitive to a temperature flux, and Austenite temperature at body temperature or a pre-defined comfortable temperature for the user, will be employed. Finally, only the strain sensor, the microcontroller, and a Bluetooth unit, fixed to the pocket and connected to as low as a 5V voltage DC supply, will be enough for the working of the *smart jacket*. The advantage of using SMAs embedded or woven as fibers/fabric of the jacket, instead of using other temperature sensors such as thermocouples, is its non-interference with any electrical equipment.

Furthermore, the 5 V battery to power the BLE and strain sensor in the jacket will have no interference with any electrical equipment in the industrial or household environment. In an industrial application using jackets with multiple users can cause a thermocouple to malfunction due to the presence of radio equipment and electric lights. Such temperature sensors also can cause false alarms due to inaccurate data, given their issue with stray voltage pickup and frequent calibration. On the other hand, not only the proposed framework is able to overcome these issues, but SMA embedded *smart jacket* can also be worn and washed regularly like a conventional jacket by simply removing the strain sensor from the button-like attachments and the micro-controller and battery unit from the pocket.

### 5.2. Use Case 2: Human-Robot Interaction: Following Leader

Many emerging robot applications in Industry 4.0 and Industry 5.0 need robots to interact with humans as a capable human-robot team. Furthermore, automation is required in Industry 4.0 in which robots collaborate [95] and interact [96] with each other [97,98]. These applications include Robots for homes, hospitals, and offices are on the horizon, but they already exist in more sophisticated situations, such as space exploration. HRI is based on a shared environment and information sharing; the leader-follower setting prohibits sophisticated social engagement. HRI has become more crucial in Industry 4.0 and Industry 5.0, particularly in robotics. Creating safe and intuitive solutions is critical as robots and humans share an increasing number of workplaces. The physical proximity of interacting partners poses the issue of integrating human behaviors into the robot’s decision-making process. The leader-follower paradigm, which entails designing a compliant robotic system that reacts to human actions and supports humans in reaching a goal, is commonly used to circumvent this difficulty. On the other hand, many ordinary interaction settings necessitate actively cooperating individuals who can independently contribute to a shared task.

Evrard and Abderrahmane [99], and Kheddar [100] established a homotropy that facilitates fluent shifts between the leader and follower roles in order to allow for a flexible role distribution with seamless transitions. The robotic system may alter its behavior based on haptic and force readings during the engagement. The leader sends the signal to the follower to perform tasks and contribute to achieving the common goal. The intention may be inferred based on the current state, velocity, and interaction forces on a motor level, as specified in Li and Ge [101]. A radial basis function neural network is used in [101] to train a function that translates the current data to the desired result. A robot is shown to follow human movements using these approximations. In this leader-follower activity, the results show that the person requires less effort than a standard impedance controller. A combination of interaction primitives may be used to mimic human movement trajectories with diverse intentions in direct collaboration contexts [102]. Following the taught interaction primitives, the robot can engage in the interaction after categorizing the present human primitive.

Being the leader of an interaction, in particular, necessitates for communication to exchange information between the leader and the interacting followers. Acting as a Follower entails adapting to a partner based not just on strong predictive abilities but also on the capacity to suppress automatic resonance in order to focus on the partner’s purpose. This supports the idea that joint actions entail a sort of communication in which smooth coordination is only possible when partners properly transmit and interpret motor signals. A coordinated complementing choreography is achieved only when both the leader and the follower accomplish their jobs well. To our best knowledge, the use of SMA is not addressed for human leader shoes to lead robots in Industry 4.0 applications. Some work on self-tightening shoes [103] and SMA insoles [104] have been presented before. Therefore, using similar design concepts, we how shoes integrated with SMA can send the human activity to robots to achieve the common goal efficiently; the authors of Li et al. [105] discussed activity recognition with challenges.

Various surroundings and activities need varying levels of autonomy, adaptability, learning ability, and conformity from the robot. We hope to develop difficulties that must be solved to enable physical HRI using SMA. We do not see the robot as a master-slave but rather as a means of demonstrating how sensorimotor contingencies might be used to produce autonomous behavior in social interaction. The recent advanced smart materials and smart wearable devices enable the collaboration of robots and humans to perform tasks effectively and efficiently. For instance, following the leader’s concept is required for collaboration and interaction, enabling robots to fellow humans in Industry 4.0 applications. The concept of the following leader can be applied to industries like mining, agriculture, construction, delivery, and logistics. Human leaders followed by robots would be associated with significant and better performance in productivity, engagement, role ambiguity, and employee satisfaction. Leader-follower behaviors often include a single unique leader interacting with their gregarious colleagues. The gregarious robots cannot accomplish the mission if the leader fails by hacking or crashing. Blockchain technology is employed as a communication tool for leaders to broadcast instructions to their followers. In addition, the blockchain provides a secure way for robots to store and retrieve shared data in a decentralized fashion.

**Figure 8:**
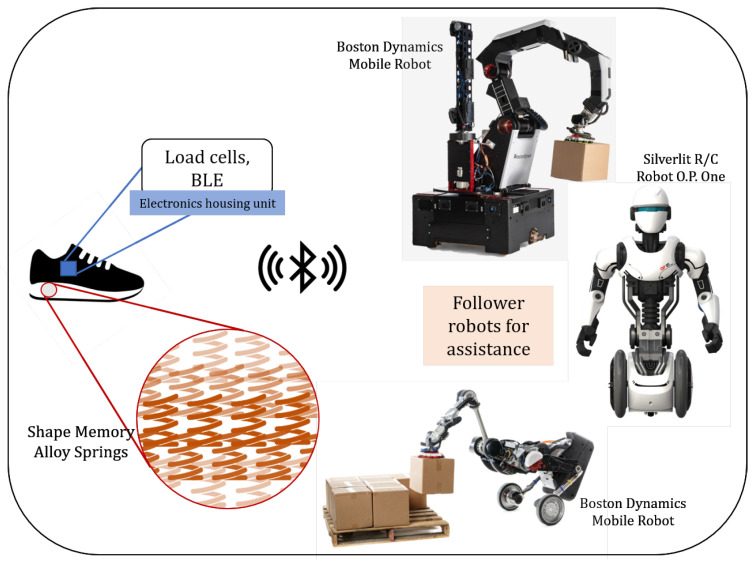
Human-Robot Interaction Use Case: Smart Shoes. Human leaders followed by robots would be associated with significant and better performance in productivity, engagement, role ambiguity, and employee satisfaction.

**Figure 9:**
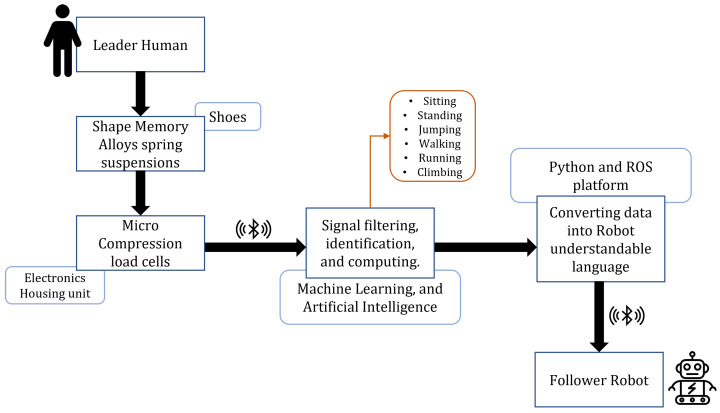
Algorithm for the HRI use case framework. The Machine Learning algorithm is essential to identify user movement conditions in real-time, especially for signal filtering and data recognition.

A critical part of the Leader-Follower robotic system is the real-time and two-way data transfer between the two parties, and to successfully achieve this, the connection must be seamless and fast. In the framework proposed, as can be seen in Figure 8, a novel SMA spring-embedded shoe sole is connected to the movement behavior of follower robots as per the user’s requirement. The *pseudoelastic* behavior of SMAs, as discussed in Section 3, allows us to utilize it under high load and subsequently high deformation conditions. Unlike the previous case, this framework does not utilize the shape memory effect; instead, the Superelastic property of high load-bearing, austenite phase SMA is considered. The SMA springs are embedded inside the shoe’s sole, and the deformation under the compressive loading due to the user’s body weight is measured using load cells. The cushioning effect from the SMA springs saves the load cell from any damage. The received load cell data is then filtered, processed, and pre-defined to identify six major activities: sitting, standing, jumping, walking, running, and climbing. This classified data is then transferred to the follower robot for behavior modeling. Machine learning can help process gathered data by SMA about human activity. The generated model can be shared with all follower robots to fulfill human activity and perform tasks collaboratively. Depending upon the state of movement of the ‘Leader Human’, the ‘Follower Robot’ is trained accordingly. The use of SMA in the shoes as a suspension provides cushioning and ensures the longevity of the product. A detailed system architecture is shown in Figure 9.

Briefly, the SMA-shoes enable a human to control swarm robots by improving the ability of robots to sense the leader’s direction, speed, stop, and velocity as it follows the leader. SMA-shoes allow humans to lead other robots, expanding the navigation range in dynamic environments (i.e., construction and civil engineering spaces requiring special training). Therefore, many applications for swarm robots follow humans and positively affect safety, search and rescue, productivity, work quality, and performance. Similar to this, research has been ongoing in the field of *smart shoes* [106], wearable wrist [107] and elbow exoskeleton [108].

### 5.3. Smart Jackets and Smart Shoes in IoT and Industry 4.0

The requirement of safety gear depending on the industrial environment has been essential since the first industrial revolution. With the advent of Industry 4.0 and the integration of smart manufacturing system lines and products, there has also been a surge in the integration of smart wearables to supervise, maintain, protect, and assist the users with the industrial environment [109]. The major application of industrial smart wearables is required to monitor, support, train, and track. From USA-based Guardhat’s Smart Helmets to SolePower’s Smart Boots, many industrial setups are now encouraging the use of smart devices for their workers [110].

While these devices are loaded with multiple sensors, including location, proximity, heat, Air Quality Index (AQI), etc., for the smooth functioning of the user, they lack a real-time interaction with the environment to create a more organic and seamless environment. The frameworks of the use cases in this article propose not only tracking and hence safety of the wearer of the jacket and the shoes but also the body temperature and the motion behavior interact with the environment and other robots, respectively, to create a coherent environment.

**Figure 10:**
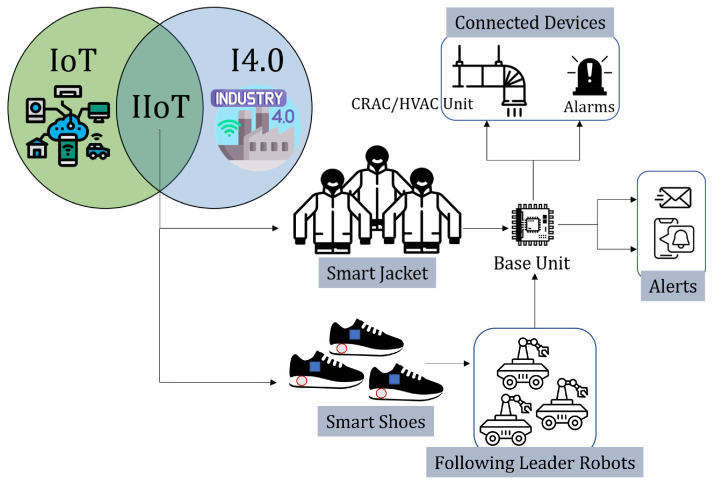
Smart Jacket and Smart Shoes in IoT and I4.0 for worker safety, protection, interaction, and detection of hazardous environment for alert and database.

The idea works beyond the human-computer and human-robot interaction and integrates itself with IIoT, as shown in Figure 10. The data obtained from the strain sensors in the jacket and the shoes are sent to the base unit, where the data is categorized and used accordingly. The temperature maintenance-related data is sent to the CRAC/HVAC unit, whereas the shoe data is shared among the follower robots, and the target robot is locked to follow. Any discrepancies in the data are analyzed and, if found crucial, are sent to the alarming units and as alerts on email and smartphones. The machine learning aspect of the smart shoe data to train a humanoid or robot motion is discussed in the previous section. For the proposed IIoT integration to be successful, it is also imperative that the *smart jacket* data is obtained from multiple users and is calibrated as per the environment they are currently placed.

## 6. Discussion

### 6.1. Related Work

In Table 1, we discuss the current state-of-the-art SMA-based smart wearables and compare their field of application based on five major criteria- Rehabilitation, Protection, Assistance, Human-Robot/Computer Interaction, and Industry 4.0. All the references are arranged in ascending order from past research to recent. This section discusses the related work in further detail and compares the proposed conceptual frameworks against the existing technologies. The application of SMA in textiles has been explored since as early as the 90s. The use of SMAs in clothing to morph upon actuation and create an air gap for insulation has been suggested by Congalton [111]. In the past decade. Stirling et al. proposed the use of SMA for an active soft orthotic that activates during the gait cycle to provide an individualized control [76]. Going forward, Holschuh-Newman developed ‘*constricting on command*’ garments [83]. The authors proposed a novel technique to integrate active materials in clothing using SMA coil actuators in modular 3D printed cartridges. Yuen et al. design an active variable stiffness fiber concept for use in fabrics to control the motion of soft robotics [112]. Apart from the fabric concepts, SMA-based exoskeletons for rehabilitation applications are becoming increasingly popular. Hope-McDaid developed a wrist and forearm exoskeleton for robotic-assisted in-home rehabilitation therapy. The authors also control the cooling rate and other factors to further sophisticate the physical and mathematical model [113]. Hwang et al. worked on a miniature haptic ring actuated and controlled by shape memory alloy wires. The design consists of a straightforward driving mechanism to deliver pressure and shearing force to the user’s fingerpad [77].

A considerable amount of research in SMA-based wearables seems to focus on the thermal shape memory effect and exoskeletons. Another work done in the field of SMA-based exoskeletons was by Copaci et al., wherein the authors designed one degree of freedom flexion-extension exoskeleton for rehabilitation and assistance, especially for patients with experienced stroke-related disability [114]. In an extension to their work, Copaci et al. further improve upon their concept and add pronation-supination degrees of freedom to the SMA-based elbow exoskeleton [82]. Chernyshov et al. devise miniature full-finger haptic rings to simulate a sense of pressure for the user. They further use nonlinear autoregressive neural networks to overcome the multiple SMA-related drawbacks which we encounter in most thermally actuated SMA actuators cases, such as delayed onset and cooling lag [78]. Kazeminasab et al. focus their work on high force exerting SMA-based arm exoskeleton in physiotherapy and object manipulation tasks [80]. Another unique method to integrate memory alloys with fabric for active clothing is to knit the fibers along with the fabric [47]. Granberry et al. introduce a circumferentially contractile self-fitting garment that conforms to the wearer’s body in response to temperature change. Another wrist exoskeleton based on SMA thermal actuation concept was proposed by Jeong et al. to assist patients with wrist motions [81]. The authors design a novel SMA actuator system to control a wrist’s flexion-extension and ulnar-radial deviation.

More recently, Villoslada et al. explored the applications of SMA-based exoskeletons for extravehicular activities [115]. The purpose of the exoskeleton is to reduce the stiffness in the EVA gloves by using pressurized gloves to counteract the forces. To integrate the memory alloy with fabric, Park-Park proposed a fabric muscle to fabricate a suit-type soft wearable robot [84]. The authors attached the SMA fabric muscle to a BOA, an adjust and lock mechanism, and utilise the shape morphing and recovery stress from thermally actuated SMA to assist the wearer in picking up heavier objects. In another work on smart clothes for ankle assistance, Kim et al. embedded SMA in stretchable fabric with anchor points and wire routing to generate an assistive force based on walking kinematics [91]. More recent research by Bartkowiak et al. discusses protective clothing for fire hazards using SMA actuators to create insulation between inner and outer fabric layers. This was done by placing an SMA element pad between two consecutive layers of clothing and actuating it to create an air gap that acts as insulation against heat [92].

**Figure 11:**
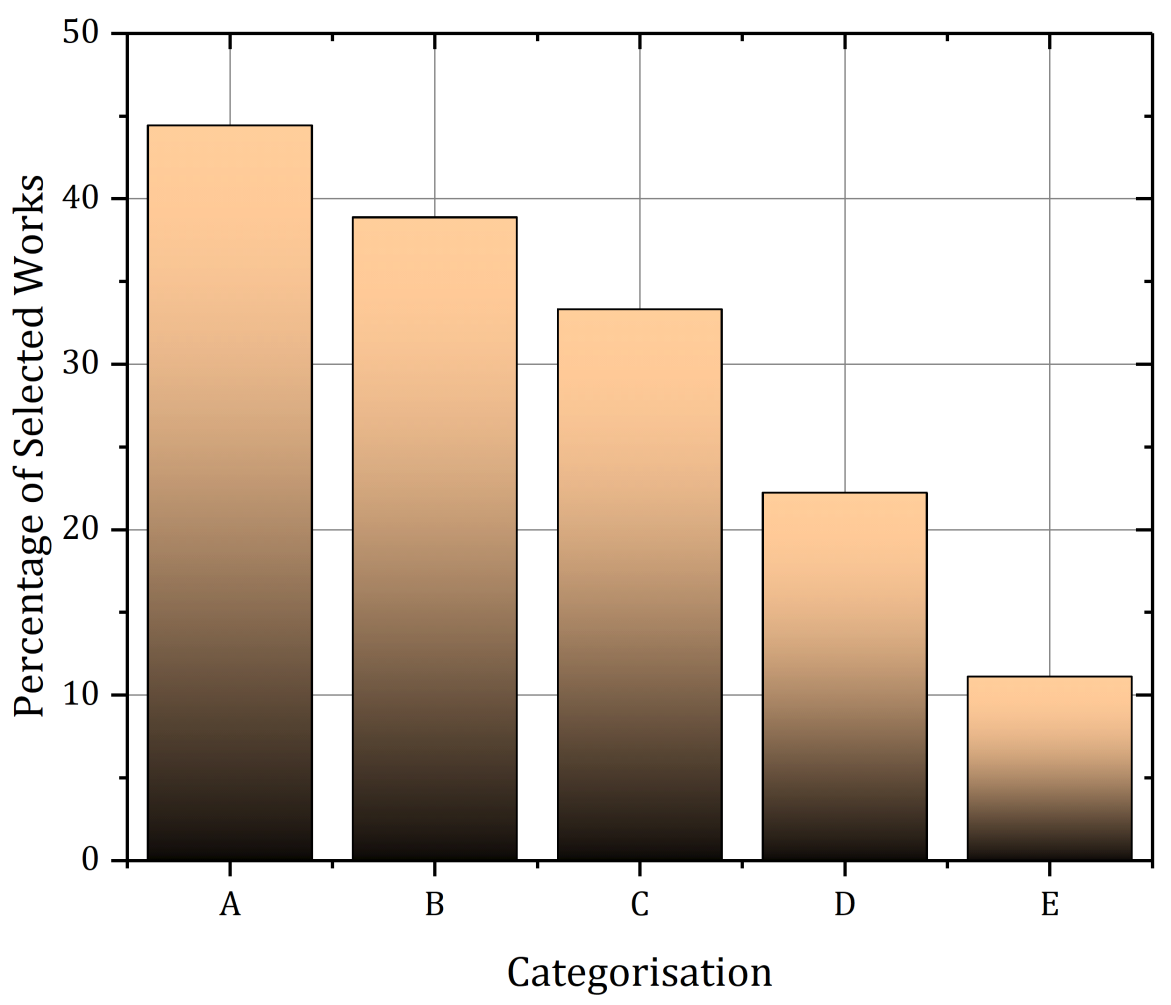
Percentage of Selected Works per Category defined for evaluation. A-Rehabilitation, B-Protection, C-Assistance, D-Human Robot/Computer Interaction, and E-Industry 4.0.

A rapidly growing interest in SMA-based smart wearables for rehabilitation and assistance is being observed. Between 2021 and 2022, multiple articles were published which had shape memory alloy-based actuators for smart textiles [116] or shoes [117] as their focal point. These researches are focused on rehabilitation [118] and assistance-based applications [119]. The first conceptual framework, *smart jacket*, talks about using SMA-embedded clothing to create a smart environment and identify and collate hazardous domains within a factory setup. This use case will not only help create a smart factory environment but also can be used for smart homes and can be extended to the smart city concept. The second conceptual framework, *smart shoes*, visualizes and proposes an algorithm for using SMA spring embedded shoes for humanoid motion training and the follower-leader concept in smart factories. The training of a humanoid can, on one hand, help improve human-robot collaboration for a smart factory setup, and on the other hand train the humanoid to learn the various patterns of human movement and become social robots. Both these use cases are compared against the most recent and related work in Table 1 for their application in various fields. A good deal of research on smart jackets and smart shoes has been conducted, however, their application as Industrial Internet of Wearable Things is still not discussed much in detail. This article aims to bridge this gap and further the use of smart wearables in IIoT environments. Figure 11 shows the percentage of related work per category of the selected works shown in Table 1.

**Table 1:** State-of-the-Art SMA wearables. Comparison of proposed use cases and categorization on the basis of application in [A]-Rehabilitation, [B]-Protection, [C]-Assistance, [D]-Human Robot/Computer Interaction, and [E]-Industry 4.0.

Ref.	Highlight	[A]	[B]	[C]	[D]	[E]
[111] (1999)	Using actuated SMA embedded in clothing to create air gap for insulation. The applications are proposed for wearable garments for fire service, drivers in enclosed vehicles, racing drivers, etc.	×	✓	×	×	×
[76] (2011)	Active soft orthotic for the knee, actively controlled by SMA springs, for gait treatment, associated with neuromuscular disorders.	✓	×	×	×	×
[83] (2016)	Novel SMA cartridge systems are fabricated to be integrated with active compression garments and envisioned for applications in space medicine, and extravehicular activity.	×	✓	×	×	×
[112] (2016)	SMA-based active variable stiffness fibers for tunable structural performance. Demonstrated motion control through surface interaction.	×	×	✓	×	×
[113] (2017)	Wrist and forearm exoskeleton for in-home robot-assisted rehabilitation therapy. Actuation modules are designed which contain SMA.	✓	×	×	×	×
[77] (2017)	Miniature SMA wire-based haptic ring to display touch and shearing force to a fingerpad. The behavior is heavily influenced by the electro-thermomechanical behavior of SMA.	×	×	×	✓	×
[114] (2017)	Medical elbow rehabilitation exoskeleton based on SMA actuators. Proposed ergonomic design solution with HRI characteristics.	✓	×	×	×	×
[78] (2018)	SMA-based wearable haptic device for silent pressure sense on the skin. Neural network-based characterisation to accurately predict device behavior.	×	×	×	✓	×
[80] (2019)	A tendon-driven hand exoskeleton used to exert extremely high forces to grasp objects in various configurations.	✓	×	×	×	×
[47] (2019)	A novel knitted NiTi-based dynamically conforming wearable fabric in response to the wearer’s temperature is introduced.	×	✓	×	×	×
[82] (2019)	Therapy exoskeleton for elbow joint based on SMA actuation. Noiseless operation increases its useability.	✓	×	×	×	×
[81] (2019)	SMA-based wearable soft robot to assist in wrist motion specifically for patients with lower arm movement difficulties.	✓	×	✓	×	×
[115] (2019)	Designed to reduce stiffness of pressurised EVA glove by giving counteracting forces based on SMA actuation.	×	×	✓	×	×
[84] (2019)	Suit-type wearable robot using shape memory alloy-based fabric muscle. The smart fabric is stitched inside the jacket along the shoulders.	×	×	✓	×	×
[91] (2020)	SMA-based soft textile for ankle plantar flexion assistance. Clothing-type wearable robot that is able to provide assistive forces without rigid kinematics.	×	✓	×	×	×
[92] (2020)	Smart textile with SMA spring elements for thermal protection. The aim was to get adaptive clothing with protection against variable work environments.	×	✓	×	×	×
Our Work 1	Human-Computer Interaction Use Case: Smart Jacket-Algorithm for the HCI use case framework. The Machine Learning algorithm is essential especially in the multi-user case to analyze and optimize the thermostat.	✓	✓	✓	✓	✓
Our Work 2	Human-Robot Interaction Use Case: Smart Shoes- Human leaders followed by robots would be associated with significant and better performance in productivity, engagement, role ambiguity, and employee satisfaction.	✓	✓	✓	✓	✓

### 6.2. Challenges and Future Trends

The major challenge envisaged with Use Case 1, *smart jacket*, will be regarding the sensitivity of the wires. The higher the sensitivity to thermal variations of the user and the environment will decide how optimized the overall system is. A lack of high sensitivity may lead to lesser collaboration between the users, and the environment may act in accordance with only one or an average of the user data. To overcome this, we propose that the jacket’s SMA fibers are calibrated so that their thermal response is captured accurately and precisely. In addition, a thorough study of the fatigue life of the SMA fibers is essential to determine the life cycle of the jacket. The user can have an informed decision based on the life cycle of the jacket and the deterioration pattern of the sensing mechanism, and its impact on the smart environment.

In the case of Use Case 2, *smart shoes*, the major challenge that the authors identify will be in the context of multiple *smart shoes* and follower-leader robots on the floor. Since the proposed mechanism does not mention the location detection of the leader, the leader must have location detection to avoid collisions. The proposed mechanism will work better in an environment where the number of follower robots is minimum. They can be further utilized during the training process of a humanoid to feed data for a human’s walking, jumping, skipping, etc., patterns. To ensure the comfort and conformability of the smart shoe, the authors suggest ergonomics and optimization studies for SMA spring placement in the shoe sole to distribute the user load evenly.

In the future, to measure the location of the proposed follower robot in Use Case 2, we will combine extremely stretchy skin patch-like sensors made utilizing a stretchable polymer and IMUs (Inertial Measurement Unit) or GPS (Global Positioning System) based on the requirement. Furthermore, the feedback supplied by the sensors may be used to detect the user’s position, thus making it mimic not only the walking but also locate the leader. This data will be optimized in an industrial application with higher footfall to avoid robot collisions while following the leader. The location data from all workers can be collated and used to analyze the unit’s efficiency, security, and save the user from a hazard. With the growing research in intention sensing technologies such as EMG (Electromyography), force, or ultra-sensitive strain measurement systems, a futuristic *smart jacket*, in Use Case 1, can be used to benefit patients who have difficulty controlling their activities of daily living by not only optimizing the environment but also integrate it with user intentions to control other aspects of the surroundings.

## 7. Conclusions

In this article, the authors have proposed two conceptual frameworks for SMA-based wearables, especially in an industrial environment or for a *smart factory* concept. The proposed use cases discuss the *smart jacket* for smart environment and habitation, whereas the *smart shoes* are envisaged to combine with follower-leader robots and also humanoid learning of multiple human walking patterns. The importance of using Shape Memory Alloy for these applications stems from the Shape Memory Effect and pseudoelastic properties of SMA, based on which phase the material is in its room temperature. While using martensite phase SMA in the jacket can act as a safer and a durable option to detect body temperature vs. environment conditions upon phase transformation and thus optimize the temperature conditions, the use of pseudoelastic/superelastic austenite phase SMA in the shoes ensures the longevity of the shoes as well as capturing the walking pattern by tracking the displacement behavior of the SMA springs. The IIoT works on a seamless combination of human-robot interaction to create a safer and more efficient environment. As proposed in this article, the *smart jacket* and *smart shoes* combined with the CRAC/HVAC unit (Computer Room Air Conditioning/Heating, Ventilation, and Air Conditioning) and the Alarms unit integrated with phone and email notifications will result in a highly secure and risk-free environment for the users. Further analysis of collected data from the *smart jacket* and the *smart shoes* can be used to identify possible hazardous and maximum footfall zones, respectively. The article also presents the various application domains of SMAs and their possible applications in several other fields. It is envisaged that through safe working conditions and integrating the proposed conceptual frameworks to IIoT, the *Smart Factory* concept moves closer to Industry 5.0. The authors propose the ‘Challenges and Future Trends’ Section as a guideline for the further scope of work in this field, specifically to make a highly sensitive and efficient smart environment for an industrial or housing setup. To summarise the conclusions and future work:

1. SMA-based *smart jacket* and *smart shoes* are proposed for IIoWT for a smart factory or Industry 4.0 concept.
2. The Use Cases are discussed in detail and previous work related to the Use Cases is also presented as they can be used as foundations for the proposed frameworks.
3. The future work will be to present the proof-of-concepts of the use cases and produce working models of the same.

## Data Availability

Not applicable.

## References

[1] Reddy D. (1976). Speech recognition by machine: A review. Proc. IEEE.

[2] Bass T.A. (1985). The Eudaemonic Pie.

[3] Bolt R.A. (1980). “Put-That-There”: Voice and Gesture at the Graphics Interface. SIGGRAPH Comput. Graph..

[4] Wu Q., Sum K., Nathan-Roberts D. How Fitness Trackers Facilitate Health Behavior Change. Proceedings of the Human Factors and Ergonomics Society Annual Meeting.

[5] Gayathri K., Easwarakumar K., Elias S. (2017). Probabilistic ontology based activity recognition in smart homes using Markov Logic Network. Knowl. Based Syst..

[6] Talukder M.S., Chiong R., Bao Y., Malik B.H. (2019). Acceptance and use predictors of fitness wearable technology and intention to recommend. Ind. Manag. Data Syst..

[7] Zheng Y., Tang N., Omar R., Hu Z., Duong T., Wang J., Wu W., Haick H. (2021). Smart Materials Enabled with Artificial Intelligence for Healthcare Wearables. Adv. Funct. Mater..

[8] Barrett M., Combs V., Su J.G., Henderson K., Tuffli M.A. (2018). AIR Louisville: Addressing Asthma With Technology, Crowdsourcing, Cross-Sector Collaboration, and Policy. Health Aff..

[9] Jenkins K., Nguyen V., Zhu R., Yang R. (2015). Piezotronic Effect: An Emerging Mechanism for Sensing Applications. Sensors.

[10] Kim J., Jung H., Kim M., Bae H., Lee Y. (2021). Conductive Polymer Composites for Soft Tactile Sensors. Macromol. Res..

[11] Kim J., Son D., Lee M., Song C., Song J.K., Koo J.H., Lee D.J., Shim H.J., Kim J.H., Lee M. (2016). A wearable multiplexed silicon nonvolatile memory array using nanocrystal charge confinement. Sci. Adv..

[12] Bhagavatheswaran E.S., Parsekar M., Das A., Le H.H., Wiessner S., Stöckelhuber K.W., Schmaucks G., Heinrich G. (2015). Construction of an Interconnected Nanostructured Carbon Black Network: Development of Highly Stretchable and Robust Elastomeric Conductors. J. Phys. Chem. C.

[13] Liu Q., Chen J., Li Y., Shi G. (2016). High-Performance Strain Sensors with Fish-Scale-Like Graphene-Sensing Layers for Full-Range Detection of Human Motions. ACS Nano.

[14] Liang J., Li L., Niu X., Yu Z., Pei Q. (2013). Elastomeric polymer light-emitting devices and displays. Nat. Photonics.

[15] Matzeu G., Naveh G.R.S., Agarwal S., Roshko J.A., Ostrovsky-Snider N.A., Napier B.S., Omenetto F.G. (2021). Functionalized Mouth-Conformable Interfaces for pH Evaluation of the Oral Cavity. Adv. Sci..

[16] Song Y., Min J., Yu Y., Wang H., Yang Y., Zhang H., Gao W. (2020). Wireless battery-free wearable sweat sensor powered by human motion. Sci. Adv..

[17] Li M., Wang L., Liu R., Li J., Zhang Q., Shi G., Li Y., Hou C., Wang H. (2021). A highly integrated sensing paper for wearable electrochemical sweat analysis. Biosens. Bioelectron..

[18] Mahmoodi S.R., Xie P., Zachs D.P., Peterson E.J., Graham R.S., Kaiser C.R.W., Lim H.H., Allen M.G., Javanmard M. (2021). Single-step label-free nanowell immunoassay accurately quantifies serum stress hormones within minutes. Sci. Adv..

[19] Lin K., Xie J., Bao Y., Ma Y., Chen L., Wang H., Xu L., Tang Y., Liu Z., Sun Z. (2022). Self-adhesive and printable tannin–graphene supramolecular aggregates for wearable potentiometric pH sensing. Electrochem. Commun..

[20] Vineetha S., Royea R., Buckman K.J., Benardis M., Holmes J., Fletcher R.L., EYK N., Acharya U.R., Ellenhorn J.D. (2020). An introduction to the Cyrcadia Breast Monitor: A wearable breast health monitoring device. Comput. Methods Programs Biomed..

[21] Sohn J.W., Kim G.W., Choi S.B. (2018). A State-of-the-Art Review on Robots and Medical Devices Using Smart Fluids and Shape Memory Alloys. Appl. Sci..

[22] Jani J.M., Leary M., Subic A., Gibson M.A. (2014). A review of shape memory alloy research, applications and opportunities. Mater. Des..

[23] Tung A.T., Park B.H., Liang D.H., Niemeyer G. (2008). Laser-machined shape memory alloy sensors for position feedback in active catheters. Sens. Actuators A Phys..

[24] Machairas T., Kontogiannis A., Karakalas A., Solomou A., Riziotis V., Saravanos D. (2018). Robust fluid-structure interaction analysis of an adaptive airfoil using shape memory alloy actuators. Smart Mater. Struct..

[25] Duerig T., Pelton A., Stockel D. (1999). An overview of NiTiNOL medical applications. Mater. Sci. Eng. A.

[26] Chang L.C., Read T.A. (1951). Plastic Deformation and Diffusionless Phase Changes in Metals—The Gold-Cadmium Beta Phase. JOM.

[27] Buehler W.J., Gilfrich J.V., Wiley R.C. (1963). Effect of Low-Temperature Phase Changes on the Mechanical Properties of Alloys near Composition TiNi. J. Appl. Phys..

[28] Bachmann F., de Oliveira R., Sigg A., Schnyder V., Delpero T., Jaehne R., Bergamini A., Michaud V., Ermanni P. (2012). Passive damping of composite blades using embedded piezoelectric modules or shape memory alloy wires: A comparative study. Smart Mater. Struct..

[29] Arrieta A.F., Bilgen O., Friswell M.I., Ermanni P. (2013). Modelling and configuration control of wing-shaped bi-stable piezoelectric composites under aerodynamic loads. Aerosp. Sci. Technol..

[30] Loughlan J., Thompson S., Smith H. (2002). Buckling control using embedded shape memory actuators and the utilisation of smart technology in future aerospace platforms. Compos. Struct..

[31] Chillara V.S.C., Headings L.M., Tsuruta R., Itakura E., Gandhi U., Dapino M.J. (2019). Shape memory alloy–actuated prestressed composites with application to morphing automotive fender skirts. J. Intell. Mater. Syst. Struct..

[32] Fonseca M., Abreu B., Goncalves F., Ferreira A., Moreira R., Oliveira M. (2013). Shape memory polyurethanes reinforced with carbon nanotubes. Compos. Struct..

[33] Bormann T., de Wild M., Beckmann F., Muller B., Goulbourne N.C., Naguib H.E. (2013). Assessing the morphology of selective laser melted NiTi-scaffolds for a three-dimensional quantification of the one-way shape memory effect. Behavior and Mechanics of Multifunctional Materials and Composites 2013.

[34] Miyazaki S. (1998). Medical and dental applications of shape memory alloys. Shape Mem. Mater..

[35] Holschuh B., Newman D. (2014). Low Spring Index, Large Displacement Shape Memory Alloy (SMA) Coil Actuators for Use in Macro- and Micro-Systems. Proc. SPIE Int. Soc. Opt. Eng..

[36] Narayanan P., Elahinia M. (2016). Control of a shape memory alloy–actuated rotary manipulator using an artificial neural network–based self-sensing technique. J. Intell. Mater. Syst. Struct..

[37] Thomas S., Almanza M., Perriard Y. Design Analysis of a Shape Memory Alloy Bias-Spring Linear Actuator. Proceedings of the 2019 12th International Symposium on Linear Drives for Industry Applications (LDIA).

[38] Bartlett M.D., Kazem N., Powell-Palm M.J., Huang X., Sun W., Malen J.A., Majidi C. (2017). High thermal conductivity in soft elastomers with elongated liquid metal inclusions. Proc. Natl. Acad. Sci. USA.

[39] Stachiv I., Sittner P., Olejnicek J., Landa M., Heller L. (2017). Exploiting NiTi shape memory alloy films in design of tunable high frequency microcantilever resonators. Appl. Phys. Lett..

[40] De Sousa V.C., Tan D., De Marqui C., Erturk A. (2018). Tunable metamaterial beam with shape memory alloy resonators: Theory and experiment. Appl. Phys. Lett..

[41] Cohades A., Michaud V. (2018). Shape memory alloys in fibre-reinforced polymer composites. Adv. Ind. Eng. Polym. Res..

[42] Meo M., Marulo F., Guida M., Russo S. (2013). Shape memory alloy hybrid composites for improved impact properties for aeronautical applications. Compos. Struct..

[43] Tao T., Liang Y.C., Taya M. (2006). Bio-inspired actuating system for swimming using shape memory alloy composites. Int. J. Autom. Comput..

[44] Bertagne C., Walgren P., Erickson L., Sheth R., Whitcomb J., Hartl D. (2018). Coupled behavior of shape memory alloy-based morphing spacecraft radiators: Experimental assessment and analysis. Smart Mater. Struct..

[45] Lee K.J., Lee J.H., Jung C.Y., Choi E. (2018). Crack-closing performance of NiTi and NiTiNb fibers in cement mortar beams using shape memory effects. Compos. Struct..

[46] Ashir M., Vorhof M., Nocke A. (2019). Influence of thickness ratio and integrated weft yarn column numbers in shape memory alloys on the deformation behavior of adaptive fiber-reinforced plastics. Compos. Struct..

[47] Granberry R., Eschen K., Holschuh B., Abel J. (2019). Functionally Graded Knitted Actuators with NiTi-Based Shape Memory Alloys for Topographically Self-Fitting Wearables. Adv. Mater. Technol..

[48] Lelieveld C., Jansen K., Teuffel P. (2016). Mechanical characterization of a shape morphing smart composite with embedded shape memory alloys in a shape memory polymer matrix. J. Intell. Mater. Syst. Struct..

[49] Bhaskar J., Sharma A.K., Bhattacharya B., Adhikari S. (2020). A review on shape memory alloy reinforced polymer composite materials and structures. Smart Mater. Struct..

[50] Hua Y., Ni Q.Q., Yamanaka A., Teramoto Y., Natsuki T. (2011). The Development of Composites with Negative Thermal Expansion Properties Using High Performance Fibers. Adv. Compos. Mater..

[51] Baz A.M., Chen T.H. (1993). Performance of Nitinol-reinforced drive shafts. Smart Struct. Mater. Smart Struct. Intell. Syst..

[52] Baz A., Imam K., McCoy J. (1990). Active vibration control of flexible beams using shape memory actuators. J. Sound Vib..

[53] Pfaeffle H.J., Lagoudas D.C., Tadjbakhsh I.G., Craig K.C. (1993). Design of flexible rods with embedded SMA actuators. Smart Struct. Mater. Smart Struct. Intell. Syst..

[54] Paine J.S.N., Rogers C.A., Smith R.A. (1995). Adaptive Composite Materials with Shape Memory Alloy Actuators for Cylinders and Pressure Vessels. J. Intell. Mater. Syst. Struct..

[55] Schetky L.M., Liang C., Rogers C.A., Hagood N.W. (1994). Hybrid composite materials using shape memory alloy actuators to provide vibration and acoustic control. Smart Structures and Materials 1994: Smart Structures and Intelligent Systems.

[56] Bidaux J.-E., Bernet N., Sarwa C., Månson J.-A.E., Gotthardt R. (1995). Vibration Frequency Control of a Polymer Beam Using Embedded Shape-Memory-Alloy Fibres. J. Phys. IV Fr..

[57] Epps J., Chandra R. (1997). Shape memory alloy actuation for active tuning of composite beams. Smart Mater. Struct..

[58] Lau K.T. (2002). Vibration characteristics of SMA composite beams with different boundary conditions. Mater. Des..

[59] Baburaj V., Matsuzaki Y. (1996). Material Damping Analysis of Smart Hybrid Composite Laminated Plate Structures. J. Intell. Mater. Syst. Struct..

[60] Kuang K.S.C., Cantwell W.J. (2003). The use of plastic optical fibres and shape memory alloys for damage assessment and damping control in composite materials. Meas. Sci. Technol..

[61] Saeedi A., Shokrieh M. (2017). Effect of shape memory alloy wires on the enhancement of fracture behavior of epoxy polymer. Polym. Test..

[62] Kirkby E., Michaud V., Månson J.A., Sottos N., White S. (2009). Performance of self-healing epoxy with microencapsulated healing agent and shape memory alloy wires. Polymer.

[63] Karimi M., Bayesteh H., Mohammadi S. (2019). An adapting cohesive approach for crack-healing analysis in SMA fiber-reinforced composites. Comput. Methods Appl. Mech. Eng..

[64] Nagai H., Oishi R. (2006). Shape memory alloys as strain sensors in composites. Smart Mater. Struct..

[65] Cui D., Song G., Li H. (2010). Modeling of the electrical resistance of shape memory alloy wires. Smart Mater. Struct..

[66] Khalili S., Dehkordi M.B., Carrera E. (2013). A nonlinear finite element model using a unified formulation for dynamic analysis of multilayer composite plate embedded with SMA wires. Compos. Struct..

[67] Hirose S., Ikuta K., Umetani Y., Morecki A., Bianchi G., Kedzior K. (1985). A New Design Method of Servo-actuators Based on the Shape Memory Effect. Theory and Practice of Robots and Manipulators.

[68] Srivastava R., Bhattacharya B. (2020). Thermoelastic and vibration response analysis of shape memory alloy reinforced active bimorph composites. Smart Mater. Struct..

[69] Srivastava R., Sharma A.K., Hait A.K., Bhattacharya B., Erturk A. (2018). Design and development of active bimorph structure for deployable space application. Active and Passive Smart Structures and Integrated Systems XII.

[70] Srivastava R., Kumar R., Bhattacharya B. Vibration Response Studies of a Bi-Morph SMA Hybrid Composite Using 3D Laser Doppler Vibrometer. Proceedings of the Smart Materials, Adaptive Structures and Intelligent Systems.

[71] Thompson S.A. (2000). An overview of nickel-titanium alloys used in dentistry. Int. Endod. J..

[72] Kilkenny N.S. (2017). Reinventing the Wheel. https://www.nasa.gov/specials/wheels/.

[73] Laurentis K.J.D., Mavroidis C. (2002). Mechanical design of a shape memory alloy actuated prosthetic hand. Technol. Health Care.

[74] Price A.D., Jnifene A., Naguib H.E. (2007). Design and control of a shape memory alloy based dexterous robot hand. Smart Mater. Struct..

[75] Bundhoo V., Haslam E., Birch B., Park E.J. (2009). A shape memory alloy-based tendon-driven actuation system for biomimetic artificial fingers, part I: Design and evaluation. Robotica.

[76] Stirling L., Yu C.H., Miller J., Hawkes E., Wood R., Goldfield E., Nagpal R. (2011). Applicability of Shape Memory Alloy Wire for an Active, Soft Orthotic. J. Mater. Eng. Perform..

[77] Hwang D., Lee J., Kim K. (2017). On the design of a miniature haptic ring for cutaneous force feedback using shape memory alloy actuators. Smart Mater. Struct..

[78] Chernyshov G., Tag B., Caremel C., Cao F., Liu G., Kunze K. Shape Memory Alloy Wire Actuators for Soft, Wearable Haptic Devices. Proceedings of the 2018 ACM International Symposium on Wearable Computers (ISWC ’18).

[79] Sabater-Navarro J., Garcia N., Ramos D., Camayo E., Vivas A. (2015). Hand neuro-rehabilitation system using Nitinol spring actuators. Robot. Auton. Syst..

[80] Kazeminasab S., Hadi A., Alipour K., Elahinia M. (2019). Force and motion control of a tendon-driven hand exoskeleton actuated by shape memory alloys. Ind. Robot. Int. J..

[81] Jeong J., Yasir I.B., Han J., Park C.H., Bok S.K., Kyung K.U. (2019). Design of Shape Memory Alloy-Based Soft Wearable Robot for Assisting Wrist Motion. Appl. Sci..

[82] Copaci D., Martín F., Moreno L., Blanco D. (2019). SMA Based Elbow Exoskeleton for Rehabilitation Therapy and Patient Evaluation. IEEE Access.

[83] Holschuh B.T., Newman D.J. (2016). Morphing Compression Garments for Space Medicine and Extravehicular Activity Using Active Materials. Aerosp. Med. Hum. Perform..

[84] Park S.J., Park C.H. (2019). Suit-type Wearable Robot Powered by Shape-memory-alloy-based Fabric Muscle. Sci. Rep..

[85] Copaci D., Serrano D., Moreno L., Blanco D. (2018). A High-Level Control Algorithm Based on sEMG Signalling for an Elbow Joint SMA Exoskeleton. Sensors.

[86] Mozer M.C. (1998). The Neural Network House: An Environment That Adapts to Its Inhabitants.

[87] Filipe L., Peres R.S., Tavares R.M. (2021). Voice-Activated Smart Home Controller Using Machine Learning. IEEE Access.

[88] Mantravadi S., Møller C., Li C., Schnyder R. (2022). Design choices for next-generation IIoT-connected MES/MOM: An empirical study on smart factories. Robot. Comput. Integr. Manuf..

[89] Zhao N., Seitinger S., Richer R., Paradiso J.A. (2021). Real-time work environment optimization using multimodal media and body sensor network. Smart Health.

[90] Zafar U., Bayhan S., Sanfilippo A. (2020). Home Energy Management System Concepts, Configurations, and Technologies for the Smart Grid. IEEE Access.

[91] Kim C., Kim G., Lee Y., Lee G., Han S., Kang D., Koo S.H., Koh J.S. (2020). Shape memory alloy actuator-embedded smart clothes for ankle assistance. Smart Mater. Struct..

[92] Bartkowiak G., Dąbrowska A., Greszta A. (2020). Development of Smart Textile Materials with Shape Memory Alloys for Application in Protective Clothing. Materials.

[93] Spini T.S., Valarelli F.P., Cançado R.H., Freitas K.M.S.D., Villarinho D.J. (2014). Transition temperature range of thermally activated nickel-titanium archwires. J. Appl. Oral Sci..

[94] Goldstein D., Jones R.E., Sery R.S. (1981). Method of Modifying the Transition Temperature Range of TiNi Base Shape Memory Alloys. U.S. Patent.

[95] Gupta A., Sundhan S., Gupta S.K., Alsamhi S., Rashid M. (2020). Collaboration of UAV and HetNet for better QoS: A comparative study. Int. J. Veh. Inf. Commun. Syst..

[96] Alsamhi S.H., Lee B., Guizani M., Kumar N., Qiao Y., Liu X. (2021). Blockchain for decentralized multi-drone to combat COVID-19 and future pandemics: Framework and proposed solutions. Trans. Emerg. Telecommun. Technol..

[97] Alsamhi S.H., Lee B. (2020). Blockchain-Empowered Multi-Robot Collaboration to Fight COVID-19 and Future Pandemics. IEEE Access.

[98] Saif A., Dimyati K., Noordin K.A., Alsamhi S.H., Hawbani A. (2021). Multi-UAV and SAR collaboration model for disaster management in B5G networks. Internet Technol. Lett..

[99] Evrard P., Kheddar A. Homotopy switching model for dyad haptic interaction in physical collaborative tasks. Proceedings of the World Haptics 2009-Third Joint EuroHaptics Conference and Symposium on Haptic Interfaces for Virtual Environment and Teleoperator Systems.

[100] Kheddar A. Human-robot haptic joint actions is an equal control-sharing approach possible?. Proceedings of the 2011 4th International Conference on Human System Interactions, HSI.

[101] Li Y., Ge S.S. (2013). Human–robot collaboration based on motion intention estimation. IEEE ASME Trans. Mech..

[102] Ewerton M., Neumann G., Lioutikov R., Amor H.B., Peters J., Maeda G. Learning multiple collaborative tasks with a mixture of interaction primitives. Proceedings of the 2015 IEEE International Conference on Robotics and Automation (ICRA).

[103] Hua W., Wang R., Li L.Y. SL 2080–Concept for Self-Tightening Shoes. https://willhua.design/sl-2080-concept-for-self-tightening-shoes.

[104] Fonte M., Palmer M. (2013). Insole and Foot Orthotics Made of Shape Memory Material (smm) Three-Dimensional Spacer Fabrics. U.S. Patent.

[105] Li Q., Gravina R., Li Y., Alsamhi S.H., Sun F., Fortino G. (2020). Multi-user activity recognition: Challenges and opportunities. Inf. Fusion.

[106] Sahal R., Alsamhi S.H., Brown K.N., O’Shea D., McCarthy C., Guizani M. (2021). Blockchain-Empowered Digital Twins Collaboration: Smart Transportation Use Case. Machines.

[107] Alsamhi S.H., Shvetsor A.V., Shvetsova S.V., Hawbani A., Guizan M., Alhartomi M.A., Ma O. (2022). Blockchain-Empowered Security and Energy Efficiency of Drone Swarm Consensus for Environment Exploration. IEEE Trans. Green Commun. Netw..

[108] Alsamhi S.H., Almalki F.A., Afghah F., Hawbani A., Shvetsov A.V., Lee B., Song H. (2021). Drones’ Edge Intelligence over Smart Environments in B5G: Blockchain and Federated Learning Synergy. IEEE Trans. Green Commun. Netw..

[109] Boyes H., Hallaq B., Cunningham J., Watson T. (2018). The industrial internet of things (IIoT): An analysis framework. Comput. Ind..

[110] Svertoka E., Saafi S., Rusu-Casandra A., Burget R., Marghescu I., Hosek J., Ometov A. (2021). Wearables for Industrial Work Safety: A Survey. Sensors.

[111] Congalton D. (1999). Shape memory alloys for use in thermally activated clothing, protection against flame and heat. Fire Mater..

[112] Yuen M.C., Bilodeau R.A., Kramer R.K. (2016). Active Variable Stiffness Fibers for Multifunctional Robotic Fabrics. IEEE Robot. Autom. Lett..

[113] Hope J., McDaid A. (2017). Development of Wearable Wrist and Forearm Exoskeleton with Shape Memory Alloy Actuators. J. Intell. Robot. Syst..

[114] Copaci D., Cano E., Moreno L., Blanco D. (2017). New Design of a Soft Robotics Wearable Elbow Exoskeleton Based on Shape Memory Alloy Wire Actuators. Appl. Bionics Biomech..

[115] Villoslada Á., Rivera C., Escudero N., Martín F., Blanco D., Moreno L. (2019). Hand Exo-Muscular System for Assisting Astronauts During Extravehicular Activities. Soft Robot..

[116] Granberry R., Barry J., Holschuh B., Abel J. (2021). Kinetically Tunable, Active Auxetic, and Variable Recruitment Active Textiles from Hierarchical Assemblies. Adv. Mater. Technol..

[117] Lee R.Z.X. An experimental investigation of Shape Memory Sock-Shoes. https://hdl.handle.net/10356/153576.

[118] Jung W.K., Lee S.M., Ahn S.H., Park J. (2022). Development and assessment of a knitted shape memory alloy-based multifunctional elbow brace. J. Ind. Text..

[119] Zhang J., Cong M., Liu D., Du Y., Ma H. (2022). A lightweight variable stiffness knee exoskeleton driven by shape memory alloy. Ind. Robot. Int. J. Robot. Res. Appl..

